# *In Vitro* Resistance Development to Nemonoxacin in *Streptococcus pneumoniae*: A Unique Profile for a Novel Nonfluorinated Quinolone

**DOI:** 10.1089/mdr.2016.0021

**Published:** 2016-10-01

**Authors:** Siddhartha Roychoudhury, Kelly Makin, Tracy Twinem, Robert Leunk, Ming Chu Hsu

**Affiliations:** ^1^ Procter & Gamble, Cincinnati, Ohio.; ^2^ TaiGen Biotechnology Co., Ltd., Taipei, Taiwan.

**Keywords:** Nemonoxacin, nonfluorinated quinolone, *S. pneumoniae*, antimicrobial resistance

## Abstract

Selection of resistant strains in *Streptococcus pneumoniae* was studied *in vitro* with nemonoxacin, a novel nonfluorinated quinolone (NFQ), in comparison with quinolone benchmarks, ciprofloxacin, garenoxacin, and gatifloxacin. In stepwise resistance selection studies, a 256-fold loss of potency was observed after three to four steps of exposure to ciprofloxacin or garenoxacin. In contrast, the loss of potency was limited to eightfold after three steps of exposure to nemonoxacin and repeated attempts to isolate highly resistant organisms after four steps of exposure yielded isolates that could not be subcultured in liquid medium. The quinolone resistance-determining regions of the target genes, *parC*, *parE*, *gyrA*, and *gyrB*, were analyzed through DNA sequencing. Known mutations, especially in the hotspots of *parC* and *gyrA*, were selected with exposure to garenoxacin, ciprofloxacin, and gatifloxacin. In contrast, mutations selected with nemonoxacin were limited to GyrA, GyrB, and ParE, sparing ParC, which is known as a key driver of resistance in clinical isolates of *S. pneumoniae*. This observation is consistent with previous data using other NFQs, which showed no loss of potency due to ParC mutations in clinical isolates. This apparently unique feature of nemonoxacin is potentially attributable to the structural uniqueness of the NFQs, distinguishing them from the fluoroquinolones that are commonly prescribed for infections by *S. pneumoniae*.

## Introduction

*S**treptococcus pneumoniae* is a major pathogen involved in serious infections, including community-acquired pneumonia, meningitis, otitis, sinusitis, and exacerbations of chronic bronchitis.^1^ While agents such as penicillin and macrolides have provided efficacious therapy for decades, their widespread use has also increased the prevalence and spread of strains that are resistant to these antibiotics.^2^ Relatively recently, fluoroquinolones have emerged as suitable therapeutic alternatives in light of their favorable potency, oral bioavailability, pharmacokinetics (PK), and pharmacodynamic (PD) profile.^3^ However, with increased clinical use, resistance to these agents is also on the rise.^4^

Fluoroquinolone resistance in *S. pneumoniae* is typically attributed to spontaneous point mutations in the quinolone resistance-determining regions (QRDRs) of the target genes *parC* and *parE* (encoding topoisomerase IV) and *gyrA* and *gyrB* (encoding DNA gyrase).^5^ While mutations in *parC* and *gyrA* have been the strongest determinant of fluoroquinolone resistance in *S. pneumoniae*, combination of stepwise mutations in all four of the target genes has rendered some of the most efficacious fluoroquinolones, such as levofloxacin and moxifloxacin, ineffective against resistant strains of pneumococci.^6^ Consequently, there is a growing unmet need for antibacterials that are able to overcome existing mechanisms of resistance and are also less likely to select new mechanisms of resistance in pneumococci.

**FIG. 1.**
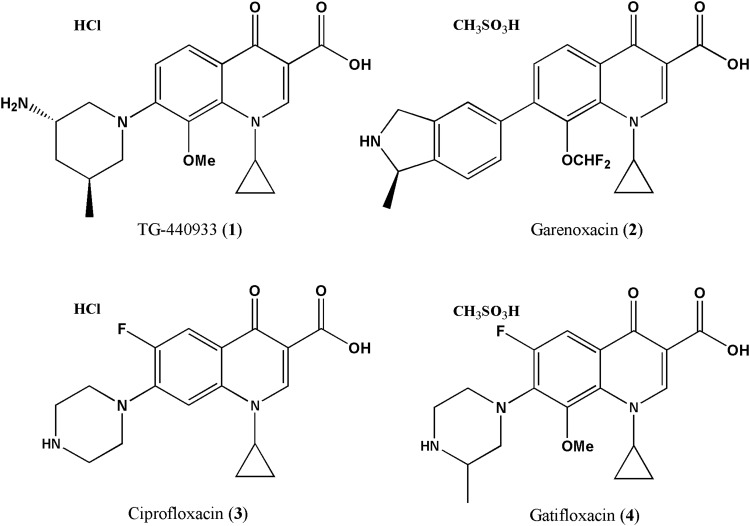
Chemical structures of nemonoxacin and other quinolones used in these studies.

In recent years, a significant number of antibacterials, including quinolones, have been designed with potent *in vitro* antibacterial activities, especially against gram-positive pathogens. However, there have been significant challenges in advancing potent new molecular entities with favorable drug-like properties, including acceptable toxicology, PK/PD, and absorption, distribution, metabolism, and excretion (ADME) profiles through clinical development. The discovery of the nonfluorinated quinolones (NFQs) deviated from this pattern in two significant ways. First, replacement of the fluorine atom in the C-6 position with hydrogen in the NFQs (Fig. 1) led to a unique structure–activity relationship (SAR) profile. The discovery strategy thus focused on identifying molecules with high potency against quinolone-resistant gram-positive pathogens, such as methicillin-resistant *Staphylococcus aureus* (MRSA) and quinolone-resistant *S. pneumoniae*. This unique approach led to the discovery NFQs, which did not exhibit loss of potency due to QRDR hotspot mutations in *parC.*^7,8^ Second, by taking a multipronged approach toward lead optimization, the NFQ lead discovery effort succeeded in identifying nemonoxacin as the clinical candidate by optimizing toxicology, PK/PD, and ADME profiles, in parallel with potency against drug-resistant gram-positive pathogens. Subsequently, nemonoxacin was shown to be clinically safe, tolerable, and efficacious in community-acquired pneumonia^9^ and diabetic foot infections.^10^ In addition, nemonoxacin has received its first regulatory approval^11^ and was granted Qualified Infectious Disease Product status by the U.S. FDA.

In this study, a stepwise resistance selection assay with *S. pneumoniae* was used to assess *in vitro* resistance development to nemonoxacin in comparison with ciprofloxacin, garenoxacin, and gatifloxacin. A markedly different resistance selection profile for nemonoxacin was revealed relative to the quinolone comparators.

## Materials and Methods

### Bacterial strains

For *in vitro* resistance selection studies, *S. pneumoniae* strain ATCC 49619 from the American Type Culture Collection (Rockville) was used. In addition, three unrelated clinical isolates of *S. pneumoniae* (941117, 944006, and 970663) were isolated from patient specimens in U.S. hospitals (2001–2002) for testing. The isolates selected represented three different geographic regions within the United States, different specimen sources, and different serotypes. *S. pneumoniae* 941117 (serotype 9C) was a bloodstream isolate from the East South Central United States*. S. pneumoniae* 944006 (serotype 6C) was a sputum isolate from the mid-Atlantic United States. *S. pneumoniae* 970663 (serotype 19C) was a nasopharynx/throat/nose isolate from the West North Central United States. All three clinical isolates were wild type at GyrA, GyrB, and ParC. *S. pneumonia*e 941117 and *S. pneumonia*e 944006 had an Ile460-Val mutation at ParE. *S. pneumoniae* 970663 was wild type at ParE.

### Reagents

Nemonoxacin, garenoxacin, ciprofloxacin, and gatifloxacin (Fig. 1) were synthesized in-house, following well-characterized procedures. Purity was >99%. The salt form of test compound (Fig. 1) was taken into account when weighing compounds for susceptibility and resistance studies. Primers for QRDR DNA sequencing^12^ were obtained from Sigma-Genosys. Platinum polymerase chain reaction (PCR) Supermix high fidelity and a low DNA mass ladder were obtained from Invitrogen. A PCR purification kit, QIAquick, from QIAgen was used. All other commercial reagents used in these studies were of highest quality available.

### Stepwise resistance studies

Stepwise resistance selection studies were conducted at 1×, 2×, and 4× minimum inhibitory concentrations (MICs) for each step of mutant selection in 100 mm Petri plates containing 20 ml Mueller–Hinton agar II supplemented with 5% sheep blood. Agar plates were prepared 24 hr before inoculating and allowed to cool at room temperature and covered by aluminum foil to reduce the light exposure. Bacterial inoculum was prepared by suspending 24-hr bacterial colonies, grown on Tryptic Soy Agar II, in saline to an optical density at 600 nm of ∼1.0. Four plates of each concentration of antibacterial agent were inoculated with 250 μl of bacterial suspension, with a total number of cells ranging from 1 × 10^9^ to 1 × 10^10^ cfu exposed to each agent. Inoculum was spread for confluent growth across the agar surface and allowed to soak into the agar before inverting plates and incubating for 48 hr at 35°C in an enriched atmosphere of 5% CO_2_. Initial inoculum size was determined by performing a standard plate count on the bacterial suspension. Colonies were counted after 24 and 48 hr of incubation. Bacterial isolates from *in vitro* selection were archived in tryptic soy broth +15% glycerol and stored at −70°C.

MICs were determined according to Clinical and Laboratory Standards Institute methods for broth microdilution testing.^13^ As control, nemonoxacin, ciprofloxacin, and garenoxacin or gatifloxacin were tested against *S. pneumoniae* ATCC 49619 in all experiments reported in Tables 1–3. Ciprofloxacin was tested against *S. pneumoniae* 970663 in all experiments, including clinical isolates. Results were considered acceptable if MICs of the benchmark compounds were within ±1 dilution from the modal MIC for the designated strain. MICs were determined in Mueller–Hinton Broth II supplemented with 2.5% lysed horse blood, repeating a minimum of three times to determine the new 1× MIC for the next step of selection. For the stepwise resistance calculation, the highest MIC for each drug at each step was used to calculate the fold increase in MIC.

**Table 1.** MICs and QRDR Mutations in *Streptococcus pneumoniae* ATCC 49619 Isolates Selected Through Stepwise Exposure to Single Agents

*Selecting agent*	*Nemonoxacin*		*Garenoxacin*	*Ciprofloxacin*	
*Experiment*	*1*	*2*	*1*	*1*	*2*
Parent	0.06^a^	0.06	0.03	0.5	0.5
Step 1	0.06–0.5^b^	0.06–0.5	0.03–0.06	2–>8	2–8
	0.12^c^	0.25^c^ (GyrA S81Y)^d^	0.06	2 (ParC S79F)	2 (ParC S79F)
Step 2	0.03–0.06	0.5–1	0.12–0.25	16–32	32
	0.06	1 (GyrA S81Y; ParE D435N)	0.25	16 (ParC S79F); GyrA S81Y)	32 (ParC S79F; GyrA S81Y)
Step 3	0.06–0.12	0.25–0.5	1–2	32–64	32–128
	0.06	0.5 (GyrA S81Y; ParE D435N)	2 (GyrA S81F; ParC S79Y)	(ParC S79F; GyrA S81Y)	(ParC S79F); GyrA S81Y)
Step 4	NV^e^	NV	4–8	NT	NT
			4 (GyrA S81L; ParC S79Y)		
Step 5	NT	NT	4–8	NT	NT
			(GyrA S81L; ParC S79Y)		

^a^ MIC values in μg/ml.

^b^ Range of MICs for four isolates, determined three times.

^c^ MIC of isolate used in next step of selection. 1×, 2×, and 4× concentrations of this MIC were used for the next step of resistance selection.

^d^ QRDR mutations identified in that isolate.

^e^ NV, not viable; isolates were not sufficiently viable in broth culture to determine MIC.

MIC, minimum inhibitory concentration; NT, not tested; QRDR, quinolone resistance-determining region.

**Table 2.** MICs and QRDR Mutations in *S. pneumonia*e ATCC 49619 Isolates Selected Through Stepwise Exposure to Various Antibacterial Agents After Exposure to Ciprofloxacin in Step 1

		*MIC (μg/ml)*		
*Selection step*	*Selecting agent*	*Nemonoxacin*	*Ciprofloxacin*	*Gatifloxacin*
Parent		0.06	0.5	0.25
Step 1	CIP^a^ [ParC (S79F)]	0.06–0.5^b^ (0.06)^c^	2–>8 (2)	0.5–4 (0.5)
Step 2	CIP/CIP [ParC (S79F); GyrA (S81Y)]	0.25–0.5 (0.5)	16–32 (16)	4–8 (4)
Step 3	CIP/CIP/CIP [ParC (S79F); GyrA (S81Y)]	0.25–0.5 (0.5)	32–64 (32)	4 (4)
Step 3	CIP/CIP/GAT [ParC (S79F); GyrA (S81Y)]	0.25–1 (1)	16–32 (32)	4–8 (8)
Step 3	CIP/CIP/nemo [ParC (S79F); GyrA (S81Y)]	0.5–2 (1)	8–32 (32)	2–8 (8)
Step 2	CIP/GAT [ParC (S79F); GyrA (S81F)]	0.5 (0.5)	16–32 (16)	4 (4)
Step 3	CIP/GAT/CIP [ParC (S79F); GyrA (S81F)]	0.25–0.5 (0.5)	32–64 (32)	4 (4)
Step 3	CIP/GAT/GAT [ParC (S79F); GyrA (S81F)]	0.5–1 (1)	16–32 (32)	4–8 (4)
Step 3	CIP/GAT/nemo [ParC (S79F); GyrA (S81F, S82P)]	1–2 (2)	16–64 (32)	2–16 (8)
Step 2	CIP/nemo [ParC (S79F)]	0.12–0.5 (0.12)	4–32 (4)	0.5–4 (1)
Step 3	CIP/nemo/CIP [ParC (S79F)]	0.12–1 (0.12)	8–32 (16)	1–8 (2)
Step 3	CIP/nemo/GAT^d^ [ParC (S79F); GyrA (S81Y)]	1 (1)	16–32 (32)	4–8 (4)
Step 3	CIP/nemo/nemo [ParC (S79F); GyrA (S81F]	0.5–2 (1)	16–32 (32)	4–8 (4)

^a^ CIP, ciprofloxacin; GAT, gatifloxacin; nemo, nemonoxacin.

^b^ Range of MICs for three to four isolates, determined three times.

^c^ Value in parenthesis is MIC of isolate used in next step of resistance selection.

^d^ These were the only strains in which QRDR mutations (shown in brackets) were detected; all other strains described here contained wild-type QRDR sequences.

**Table 3.** MICs and QRDR Mutations in *S. pneumonia*e ATCC 49619 Isolates Selected Through Stepwise Exposure to Various Antibacterial Agents After Exposure to Nemonoxacin in Step 1

		*MIC (μg/ml)*		
*Selection step*	*Selecting agent*	*Nemonoxacin*	*Ciprofloxacin*	*Gatifloxacin*
Parent		0.06	0.5	0.25
Step 1	Nemo^a^	0.06–0.5^b^ (0.12)^c^	0.5–2 (1)	0.25–2 (0.5)
Step 2	Nemo/CIP	0.03–0.06 (0.06)	0.25–1 (0.5)	0.12–0.25 (0.25)
Step 3	Nemo/CIP/CIP	NV^d^	NV	NV
Step 3	Nemo/CIP/GAT	0.03–0.12 (0.06)	0.25–1 (1)	0.06–0.25 (0.25)
Step 3	Nemo/CIP/nemo	0.03–0.06 (0.06)	0.25–1 (0.5)	0.06–0.25 (0.25)
Step 2	Nemo/GAT	0.06 (0.06)	1 (1)	0.25–0.5 (0.5)
Step 3	Nemo/GAT/CIP	0.12–0.5 (0.12)	2–4 (4)	0.25–0.5 (0.5)
Step 3	Nemo/GAT/GAT^e^ [GyrA (S81-Y)]	0.06–0.25 (0.25)	2–4 (2)	0.5–1 (0.5)
Step 3	Nemo/GAT/nemo	0.12–0.25 (0.12)	2–4 (2)	0.25–1 (0.5)
Step 2	Nemo/nemo	0.03–0.06 (0.06)	0.5–1 (0.5)	0.25 (0.25)
Step 3	Nemo/nemo/CIP	0.06–0.12 (0.12)	1–4 (2)	0.25–0.5 (0.25)
Step 3	Nemo/nemo/GAT^e^ [ParC (S79-Y)]	0.06–0.12 (0.12)	2–4 (2)	0.25–0.5 (0.5)
Step 3	Nemo/nemo/nemo	0.06–0.12 (0.06)	0.5–1 (1)	0.12–0.5 (0.25)
Step 4	Nemo/nemo/nemo/nemo	NV	NV	NV

^a^ nemo, nemonoxacin; CIP, ciprofloxacin; GAT, gatifloxacin.

^b^ Range of MICs for three to four isolates, determined three times.

^c^ Value in parenthesis is MIC of isolate used in next step of resistance selection.

^d^ NV, not viable; isolates were not sufficiently viable in broth culture to determine MIC.

^e^ These were the only strains in which QRDR mutations (shown in brackets) were detected; all other strains described here contained wild-type QRDR sequences.

The contribution of efflux mechanisms to loss of potency after exposure to antibacterial agents was evaluated by determining MICs in the presence and absence of 10 μg/ml reserpine.^14^

### DNA sequencing

For sequencing the QRDR of gyrase and topoisomerase genes, bacterial DNA was prepared by suspending one colony of each isolate in 100 μl TE buffer (10 mM Tris.HCl, pH 8.0, and 1 mM EDTA). PCR amplification mixtures contained 90 μl high fidelity Supermix, 4 μl of the backward and forward primers, and 4 μl of DNA template. PCR consisted of preheating at 94°C for 5 min, 30 cycles at 94°C for 40 sec, 55°C for 40 sec, 72°C for 1 min, and a final incubation at 72°C for 5 min. Amplified DNA was purified using the QIAgen QIAquick spin column method and quantified by running DNA beside a low mass ladder in a 1% agarose gel. Sequencing was conducted on an ABI Prism 3100 Genetic analyzer (Applied Biosystems).

DNA sequencing of the QRDRs of *gyrA*, *gyrB*, *parC*, and *parE* was performed on two isolates of each strain with elevated MICs after each step of selection. One isolate from the two genetically characterized isolates was chosen for repeat exposure to antibacterial agents in the second step of resistance selection. Isolates selected for further steps of resistance selection were chosen on the basis of having the most genotypic changes in the QRDR sequence. Similarly, an isolate after the second step of resistance selection was chosen for repeat exposure to antibacterial agents in step 3. Ciprofloxacin was included as a control in all experiments. Sequence fragments were compared with the complete sequence of *S. pneumoniae* R6 through a BLAST search. Mutations were identified visually and compared with hotspot mutations described in the literature.

## Results

### Stepwise selection of mutants using single agents

The parent strain of *S. pneumoniae* ATCC 49619 was exposed in a stepwise manner to single agents, nemonoxacin, garenoxacin, or ciprofloxacin. Colonies growing at 4× MIC were selected for MIC determination and QRDR DNA sequence analysis of *gyrA*, *gyrB*, *parC*, and *parE* genes, and then used for the next step of resistance selection. Results are summarized in Table 1.

In experiment 1, after exposure to nemonoxacin in step 1, a one to eightfold loss of potency was observed with resultant MICs of 0.06–0.5 μg/ml. The QRDR sequences were analyzed for an isolate with an MIC of 0.12 μg/ml and this isolate retained all wild-type sequences. This isolate was used for additional steps of resistance selection. Steps 2 and 3 did not lead to any incremental loss of potency or mutations in the QRDR of target genes. Colonies observed in the fourth step of resistance selection could not be subcultured into liquid media for MIC determination. Most of these colonies were smaller and did not display the typical morphology of the parental strain.

In experiment 2 with nemonoxacin (Table 1), a similar loss of potency was observed after step 1 as in experiment 1, with resultant MICs of 0.06–0.5 μg/ml. After step 1 of experiment 2, target gene QRDR sequences were analyzed in an isolate with an MIC of 0.25 μg/ml and the GyrA hotspot had a Ser81-Tyr mutation. This isolate was used for subsequent steps of resistance selection. In step 2, a further two to fourfold loss of potency was observed, with resultant MICs of 0.5–1.0 μg/ml. Target gene QRDR sequences were analyzed in an isolate with an MIC of 1 μg/ml and this isolate harbored the previously described^15^ Asp435-Asn mutation in ParE, in addition to the previous GyrA Ser81-Tyr mutation. The ParE (Asp435-Asn) mutation was not observed in any of the mutants selected with other agents or selected with nemonoxacin subsequent to selection with ciprofloxacin or gatifloxacin in this study. This isolate was used for step 3 of resistance selection.

After step 3 of resistance selection, no incremental loss of potency was observed with resultant MICs of 0.25–0.5 μg/ml and no additional mutations in the QRDR target genes were identified relative to the parent strain. An isolate with an MIC of 0.5 μg/ml was used for step 4 of resistance selection. Similar to experiment 1, colonies observed in step 4 could not be subcultured into liquid media for MIC determination. Interestingly, with nemonoxacin, no mutations were identified in the QRDR of ParC in either of these two experiments.

**FIG. 2.**
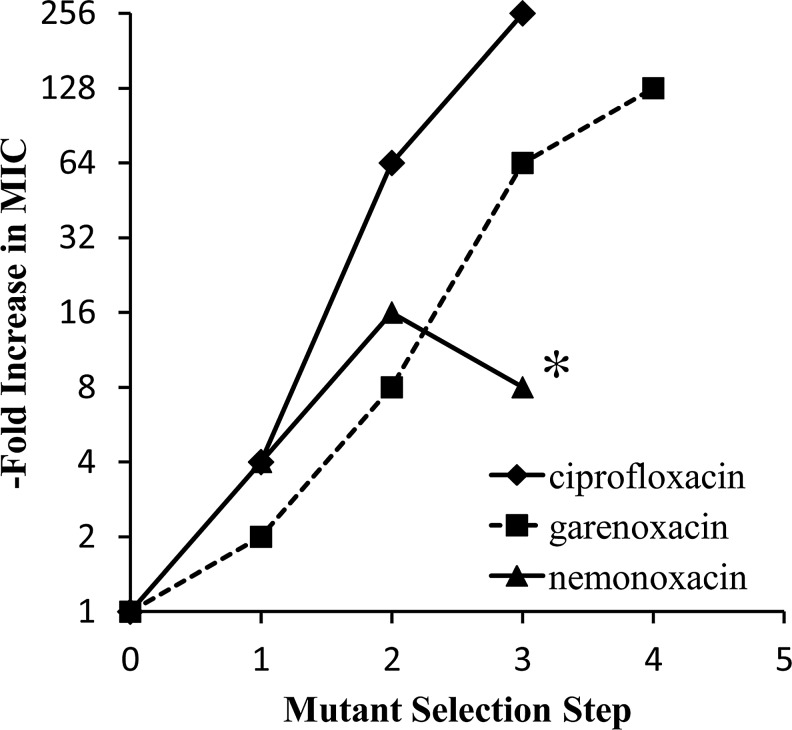
Loss of potency against *Streptococcus pneumoniae* with stepwise exposure to ciprofloxacin, garenoxacin, and nemonoxacin. Results shown are based upon modal MICs. Data for ciprofloxacin and nemonoxacin are from Table 1, experiment 2, where the increase in resistance was greater for both agents. *MIC of Step 4 mutant colonies selected on nemonoxacin could not be determined as mutants could not be subcultured into liquid media. MIC, minimum inhibitory concentration.

The stepwise selection of resistant mutants was also completed with two comparators, garenoxacin and ciprofloxacin (Table 1). With garenoxacin, a 32 to 256-fold loss of potency was observed in three to five steps of resistance selection. Known QRDR hotspot mutations in both GyrA and ParC were selected with garenoxacin after step 5 [MIC 8 μg/ml; GyrA (Ser81-Phe or Leu); ParC (Ser79-Tyr)]. With ciprofloxacin, a 64 to 256-fold loss of potency was observed in three steps of resistance selection. Known QRDR hotspot mutations in both ParC and Gyr A were selected with ciprofloxacin after step 3 [MIC 128 μg/ml; ParC (S79F); GyrA (S81Y)]. The relative loss of potency in stepwise resistance selection with all three agents is shown in Figure 2.

### Stepwise selection of mutants using multiple agents

In additional stepwise *in vitro* resistance selection experiments, isolates of *S. pneumoniae* ATCC 49619 exposed to ciprofloxacin in step 1 were subsequently exposed to ciprofloxacin, gatifloxacin, or nemonoxacin. Results are shown in Table 2. Consistent with previously published data,^16^ exposure to ciprofloxacin led to the selection of step 1 mutants with the QRDR hotspot mutation in ParC (Ser79-Phe). Subsequent exposure to ciprofloxacin or gatifloxacin resulted in a second hotspot mutation in GyrA (Ser81-Tyr or Phe) with further loss of potency and MICs of 16–32 μg/ml, leading to high-level resistance. Exposure to nemonoxacin in step 3, led to the isolation of a mutation in the Ser-82 residue of GyrA, subsequent to the one in Ser-81 isolated in step 2 with exposure to gatifloxacin. This relatively rare mutation in *S. pneumoniae* GyrA was associated with a twofold incremental loss of potency of nemonoxacin and a final MIC of 1 μg/ml. Consistent with previously published results using other NFQs,^17^ the ParC Ser79-Phe hotspot mutation did not reduce the potency of nemonoxacin (MIC 0.06 μg/ml), nor was any novel mutation detected in the ParC QRDR during this experiment.

Isolates of *S. pneumoniae* ATCC 49619 exposed to nemonoxacin in step 1 were subsequently exposed to nemonoxacin, ciprofloxacin, or gatifloxacin in additional steps of resistance selection. Results are shown in Table 3. No mutations in the target gene QRDR were identified in steps 1 and 2, and the loss of potency was limited to twofold for all three agents. After exposure to gatifloxacin in step 3, GyrA (Ser81-Tyr) and ParC (Ser79-Tyr) hotspot mutations were identified (gatifloxacin and nemonoxacin being the selecting agents for step 2, respectively). No mutants with a substantial increase in MIC of nemonoxacin could be selected after three or four steps of exposure to this agent (Table 3).

### Resistance selection in clinical isolates

To assess the potential for emergence of resistance in clinical isolates of *S. pneumoniae*, three distinct clinical isolates were exposed to nemonoxacin. One of the three isolates, *S. pneumoniae* 970663, was wild type for the QRDR sequences of *gyrA*, *gyrB*, *parC*, and *parE*. The other two isolates, *S. pneumoniae* 944006 and *S. pneumoniae* 941117, contained the Ile460-Val mutation in ParE. Nemonoxacin MICs were 0.03–0.06 μg/ml for all three isolates. In three steps of exposure to nemonoxacin, the overall loss of potency was two to eightfold, with resultant MICs of 0.06–0.5 μg/ml. No new mutations in the QRDR of the target genes were identified in step 1 and step 2 isolates of *S. pneumoniae* 941117 and *S. pneumoniae* 944006. After step 1 exposure of wild-type *S. pneumoniae* 970663 to nemonoxacin, a Ser81-Tyr mutation in GyrA was identified, with a resultant MIC of 0.25 μg/ml. While no new mutations were identified with this isolate after step 2 exposure to nemonoxacin, an isolate was identified after step 3 exposure with two new mutations, Ser494-Thr in GyrB and Pro454-Ser in ParE, with a nemonoxacin MIC of 0.5 μg/ml.

### Role of efflux mechanisms in resistance

The presence of efflux mechanisms was assessed by determining MICs in the presence and absence of reserpine. Involvement of efflux in resistance was considered to be evident with a decrease in MIC of at least fourfold in the presence of reserpine in the final-step mutants. No such changes in the susceptibility were observed in any of the mutants isolated in this study (data not shown).

## Discussion

The novel SAR approach toward the NFQ lead identification led to a unique potency profile against gram-positive pathogens. Previously reported studies^7^ demonstrated the relatively high potency of the NFQs, combined with low potential for *in vitro* resistance development, in *S. aureus* and MRSA. In *S. aureus*, unique point mutations (Ser52-Arg; His103-Tyr) in GrlA were required for high-level resistance development.^18^ This unique phenomenon was explained by a simultaneous dual-target inhibition model based on the overlapping effective target inhibition (ETI) concentration ranges for the two molecular targets of quinolones, DNA gyrase, and topoisomerase IV in *S. aureus.*^7,18^ At bacterial growth inhibitory or bactericidal concentrations, typical quinolones utilize one effective target at a time. In *S. aureus*, topoisomerase IV is the usual initial effective target (commonly known as the primary target) because its ETI concentration range is considered to be lower than that of DNA gyrase (also known as the secondary target). This phenomenon leads to the selection of first-step point mutations in the genes encoding topoisomerase IV, such as *grlA* in *S. aureus*. Once the *grlA* hotspot is mutated, the ETI concentration range of DNA gyrase is believed to be lower than that of the mutated topoisomerase, leading to the selection of second-step mutations in genes encoding DNA gyrase, such as *gyrA*. NFQs were shown to inhibit both targets with overlapping ETI concentration ranges, thereby lowering the potential for resistance development through stepwise mutations in one target at a time. In addition, the selection of novel mutations in GrlA suggested a unique mode of interaction of NFQs with topoisomerase IV at the molecular level in *S. aureus.*^18^

In *S. pneumoniae*, depending on the quinolone, either topoisomerase IV or DNA gyrase can be the primary target.^16^ Point mutations in topoisomerase IV are selected by certain quinolones (*e.g.*, ciprofloxacin) as first-step mutations, while DNA gyrase mutations are selected as first-step mutations by others (*e.g.*, gatifloxacin). Previous data^17^ as well as those generated in this study suggest that for NFQs, DNA gyrase is the primary target in *S. pneumoniae*, presumably with a lower initial ETI concentration range than that for topoisomerase IV. Thus, first-step mutations were selected by nemonoxacin in the QRDR of *gyrA* (Table 1). A second-step mutation was selected by nemonoxacin in ParE (Asp435-Asn) (Table 1), suggesting that topoisomerase IV is the secondary target. This observation could potentially be attributable to the structural uniqueness of nemonoxacin that allows for the selection of less frequently observed mutations in the topoisomerase IV ParE subunit in the absence of the more frequently observed mutations in its ParC subunit. However, unlike quinolones commonly used against *S. pneumoniae* infections such as moxifloxacin,^19^ no mutations in the QRDR of *parC* were selected with nemonoxacin in the current studies.

Additional mutations were selected by nemonoxacin in clinical isolates of *S. pneumoniae*, including GyrB (Ser494-Thr), ParE (Pro454-Ser), and GyrA (Ser82-Pro) (Table 2). The GyrA (Ser82-Pro) mutation is apparently a novel mutation in *S. pneumoniae*. While the GyrA (Ser82-Pro) mutation appears to provide incremental resistance in the strain with the existing GyrA (Ser81-Phe) mutation, the independent role of the Ser82 residue in resistance development remains poorly understood. The corresponding mutation (Ser85-Pro) has been reported in *S. aureus.*^20^ In the current studies, multiple attempts with different experimental methods and different isolates of *S. pneumoniae* failed to select QRDR mutations in *parC* with nemonoxacin. These results are consistent with previous data obtained with previous NFQs whose potencies were unaffected by *parC* QRDR mutations in both clinical isolates and laboratory-derived mutant strains of *S. pneumoniae.*^17^

Taken together, these findings suggest that the unique structure of nemonoxacin triggers a unique interaction with its molecular targets in *S. pneumoniae*, distinguishing nemonoxacin from known quinolones. Selection of two different second-step mutations in *parE*, following selection of a first-step mutation in *gyrA*, clearly indicates that potency of nemonoxacin against the GyrA hotspot-mutated *S. pneumoniae* is based, at least in part, on its ETI concentration range for topoisomerase IV. However, mutation in the *parC* QRDR does not appear to provide a significant mechanism for further resistance development to nemonoxacin. A plausible explanation for this apparently unique phenomenon is that nemonoxacin avoids contact with the ParC hotspots in its molecular interaction with *S. pneumoniae* topoisomerase IV. In this scenario, hotspot mutations in *parC* QRDR would not provide a selective advantage for *S. pneumoniae* against NFQs and these mutations would not lower NFQ potency. The unique structure of nemonoxacin appears to result in a unique mode of molecular interaction with the target enzymes, thereby limiting the potential for resistance development.

While other mechanisms of resistance development were not studied in detail for the NFQs, the role of efflux pumps was assessed based on MICs with and without reserpine (10 μg/ml) in the current studies. A lack of effect of reserpine on susceptibility of the mutants reduces the likelihood that efflux pumps play a major role in resistance. However, a possible role for the ABC class of multidrug transporters and other mechanisms of resistance development cannot be ruled out.

Given the widespread exposure of bacteria to ciprofloxacin and other fluoroquinolones associated with their extensive clinical and veterinary use, QRDR mutations in *S. pneumoniae* ParC are on the rise.^5^ Full retention of NFQ potency against ParC mutants (18; current studies) augurs well for the clinical success of nemonoxacin. While isolation of fourth or fifth-step mutants with progressive loss of potency to other quinolones, including garenoxacin, was associated with incremental mutations in the QRDR hotspots of the target enzymes, exposure to nemonoxacin in four steps yielded isolates that could not be subcultured for growth in liquid media (Fig. 1 and Table 1). This apparently unique phenomenon could be attributed to a high fitness cost associated with mutations necessary for high-level resistance to nemonoxacin, resulting in compromised viability.

Data from these experiments and the proposed model of target interactions suggest that clinical exposure to nemonoxacin could be qualitatively different from exposure to other quinolones with respect to the potential for developing stepwise high-level resistance in *S. pneumoniae*. While resistance development through other, yet unknown, mechanisms cannot be ruled out, data generated in this study suggest that nemonoxacin is less vulnerable to a key driver of quinolone resistance in *S. pneumoniae*, that is, QRDR point mutations in the target genes.
